# Supplementary figures and images for: Low Galectin-3 Expression Level in Primary Tumors Is Associated with Metastasis in T1 Lung Adenocarcinoma
Source: J Clin Med. 2020 Jun 25;9(6):1990. doi: 10.3390/jcm9061990 (PMC7355842; doi:10.3390/jcm9061990)

## Supplementary Materials

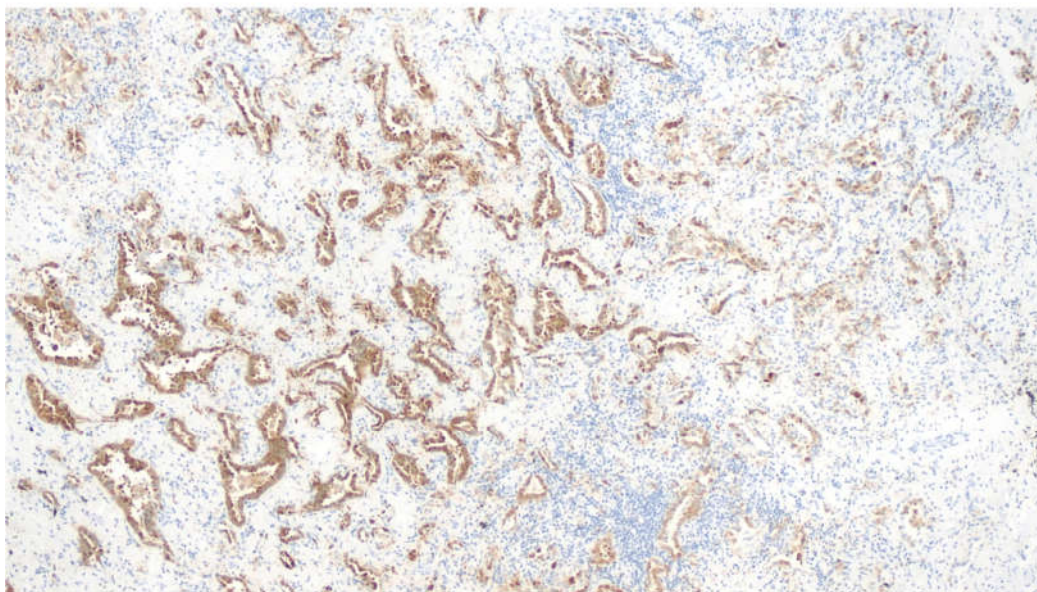

**Figure S1.** Intratumoral heterogeneity of galectin-3 expression, 40 ×.

Supplement: Supplementary file 1 [file jcm-09-01990-s001.pdf]
